# Generalized Isometric Sustained Contraction During Apnea Combined with Relaxation for Anxiety: A First Evaluation

**DOI:** 10.2174/0117450179455271260220082810

**Published:** 2026-03-16

**Authors:** Carlo Cianchetti, Mauro Giovanni Carta, Alessandra Perra

**Affiliations:** 1 Former Professor of Child and Adolescent Neurology and Psychiatry, University of Cagliari, Cagliari, Italy; 2 Department of Medical Sciences and Public Health, University of Cagliari, Cagliari, Italy

**Keywords:** Anxiety, Tension, Anti-anxiety somatic methods, Anti-anxiety maneuvers, Generalized contraction, Relaxation, (Progressive) Neuromuscular Relaxation, Autogenic training

## Abstract

**Introduction:**

There are several somatic techniques for reducing anxiety through relaxation, *i.e.*, the reduction of tension. Tension is an important component and corresponds, in the primordial mechanism of fear, to the pre-encounter immobilizing alert state of the defense cascade. It is hypothesized that an energetic generalized muscular activation, such as that used to face a fighting (circa-strike), can correct the immobilizing tension and counteract anxiety.

**Objective:**

To evaluate the degree of effectiveness against anxiety and anxiety attacks of a technique based on a phase of sustained, generalized muscular activation followed by relaxation.

**Methods:**

During a private outpatient clinical practice, selected subjects suffering from various anxiety disorders were offered the opportunity to try a technique for controlling anxiety attacks. They were instructed to activate a generalized isometric sustained contraction in apnea (GISCA), followed by a generalized relaxation (GISCA+relax), to be implemented as needed during an anxiety attack. The subjects were asked to apply the maneuver in alternation, in different episodes, with a generalized relaxation. They were asked to evaluate, after repeated use, the following aspects: a) whether GISCA+relax helped in controlling anxiety, b) if the addition of the GISCA phase was more effective than generalized relaxation alone, and c) if the GISCA phase facilitated the acquisition of the subsequent generalized relaxation.

**Results:**

The results concern the first 20 patients who were allowed a complete evaluation. A substantial majority of them (75%) reported that the complete maneuver (GISCA+relax) helped in controlling anxiety and anxiety attacks, and that was more effective than generalized relaxation alone and facilitated its acquisition.

**Discussion:**

Compared to relaxation techniques, GISCA+relax offers the advantage of easier application in acute situations, such as during an anxiety attack or in anticipation of anxiety-provoking events (*e.g.*, performance anxiety). However, a sustained generalized contraction is physical exercise, with the positive effects of any non-traumatic physical activity on health-related fitness.

**Conclusion:**

These first data, although on a limited number of patients, suggest that the proposed maneuver can help control anxiety. This new somatic behavioral approach likely counteracts anxiety by activating a primordial defense mechanism. Since it can be acquired by patients with sufficient simplicity, it is suggested that it could be considered an alternative to other somatic techniques or the use of as-needed medications.

## 1 INTRODUCTION

The main target of the non-pharmacological therapeutic intervention for anxiety is the correction of the mental mechanisms that support it, for example, with cognitive techniques. The action on the somatic expressions of anxiety is an adjuvant, at least momentarily, in therapy, which, however, has the advantage of being easier and quicker to implement.

The most widespread somatic interventions against anxiety target muscle tension, an important somatic expression of it [1-3]. This applies especially to generalized anxiety, since the most distinguishing somatic symptom of generalized anxiety disorders (GAD), compared to other anxiety disorders, is muscle tension [4]. Several relaxation methods have been developed, starting with the original Progressive Muscle Relaxation (PMR) [5] and extending to the many variations of it [6-11]. PMR involves, before relaxation, a short phase (a few seconds) of light muscle contraction, sequentially applied to different muscle groups [5, 12]. The onset of the relaxing effect is slow, and muscle relaxation is not easily achieved during a state of anxiety, particularly if it is acute and intense.

From a neuroevolutionary perspective, anxiety is the transformation of the primordial emotion, fear [13-17]. The DSM-5 [18] defines fear as “the emotional response to a real or perceived imminent threat, whereas anxiety is the anticipation of a future threat.” The anticipation of temporally uncertain and certain threat recruits broadly similar neural systems [19].

Primordial fear in the face of danger triggers an immediate reaction of the defense mechanism, based on the activation of the alarm system and the contraction of the muscles that prepare the individual for aggression or escape [20-23].

In contrast, anxiety arises in the absence of immediate danger, but with a “potential” danger, imagined or perceived as near or future, often unidentifiable. Anxiety thus represents a more complex form of fear that has evolved to enable advance preparation and response to potential, even if not immediate, threats. Therefore, anxiety does not stimulate a motor defense reaction (counter-aggression), but halts at the heightened arousal alert mechanism [24], which involves a sub-liminal contraction (a “tension”) that becomes persistent and does not discharge energy.

Therefore, anxiety, since it is not determined by events or images or ideas that can be faced, or from which one can escape, tends to be inhibitory. It carries prolonged arousal and a strong sensation of discomfort-tension with predominantly vegetative activation. Unlike the acute fear response, anxiety does not activate the muscle groups involved in defense or escape, resulting in a state of immobility and inability to react. It involves arrest with dyspnea or hyperpnea, tachycardia, increased intestinal motility, dry mouth, sweating, tension, or, on the contrary, aimless hypermotility, but not a strong defensive muscle contraction.

It is hypothesized that an active and intense contraction, similar to that used to face a fight, can correct the immobilizing tension (an incomplete, ineffective, and harmful muscular contraction) or hyper-motility, *i.e.*, motor agitation (which is likewise an incomplete, ineffective, and harmful contraction) caused by anxiety. It is also hypothesized that the effort and the consequent muscle fatigue may promote a subsequent generalized relaxation.

To achieve a sustained active contraction, *i.e.*, without interruptions for a sufficiently long period, it seems obvious to use an isometric contraction. To obtain the maximum effect, comparable to that of defense against aggression, the contraction must be quite prolonged and involve a large number of muscles, and thus be as generalized as possible, isometrically involving agonists and antagonists. A simultaneous inspiratory apnea during the sustained effort facilitates the maintenance of the contraction and counteracts the typical condition of dyspnea-hyperpnea caused by anxiety.

It is emphasized that the maneuver involves a sustained, intense contraction, reminiscent of the primordial defense mechanism of resistance. This is very different from the classic relaxation technique, which involves an initial gentle muscle contraction aimed at making the user perceive the difference between a contracted and a relaxed muscle, thus facilitating relaxation.

Based on personal positive experiences with performance anxiety, it was decided to verify whether this type of intervention could also be useful for patients. Always based on personal experience, a type of intervention was proposed as follows:

1st phase: deep inspiration, followed by isometric contraction of as many somatic muscles as possible (“generalized”), sustained for several seconds (at least 15–20 in the training phases, with the aim of gradually reaching 30–40 seconds) with maintenance of a state of apnea (GISCA).

2nd phase: subsequent generalized relaxation with deep and slow breathing.

After an adequate period of relaxation and deep breathing (30–90 sec), the generalized sustained isometric muscle contraction is again performed. The cycle is repeated until some control over the anxiety is achieved.

The aim of the study is to evaluate the effects of the maneuver during moments of anxiety, and in particular, anxiety exacerbations, regardless of the type of pathology involved and the type of pharmacological treatment in progress. This was done in outpatient settings, *i.e.*, in patients’ normal living environment, and in a within-patient cross-over comparison with the traditional relaxation maneuver.

If the results are positive, the maneuver could be an alternative to relaxation or to a medication, such as a benzodiazepine, for occasional use (as needed) during moments of anxiety.

The effects of the use of this technique in a first exploratory series of patients are presented.

## 2 METHODS

### 2.1 Study Design

Non-pharmacological therapeutic clinical feasibility pilot study comparing two treatments in acute conditions (as-needed), using repeated measures, a cross-over design, and a single-arm within-subject comparison with patient-reported outcomes.

### 2.2 Subjects

Patients receiving outpatient care at the principal investigator's private practice were recruited. Eligible patients will have anxiety disorders or other psychiatric conditions with a prominent anxiety component.

#### 2.2.1 Inclusion Criteria

- Individuals diagnosed with anxiety disorders or presenting anxiety symptoms in the context of other psychiatric disorders.
- No restriction on gender.
- Age 18 years or older.

#### 2.2.2 Exclusion Criteria

- Patients with cardiac problems or resting heart rate above 90 beats per minute, systolic blood pressure above 130 mmHg, or diastolic blood pressure above 90 mmHg.
- Patients with respiratory problems.
- Patients appearing physically unfit for exertion.

Therefore, blood pressure, heart rate, and oxygen saturation were monitored before, during, and immediately after the generalized contraction maneuver. Subsequently, patients were not asked to measure vital signs during the maneuvers.

Consecutive patients providing informed consent formed the experimental group. A separate control group was not required, as each participant served as its own control according to the within-subjects experimental design.

Patients were advised to use the somatic technique as a further as-needed therapeutic aid during anxiety episodes, in addition to their ongoing pharmacological therapy, after providing their consent to test it.

The effects of applying the technique were evaluated during a subsequent visit or by telephone contact.

Each patient, during the visit, was educated and trained to apply the proposed technique. The instructions provided, adapted as much as possible to the patient's level of understanding, are shown in the box “The maneuver,” where the description includes terms that, in practical patient instruction, were replaced by contacts or examples of maneuvers to facilitate understanding and implementation.

This report includes the first 20 patients (14 females, 8 males) from whom sufficient data for an evaluation were obtained.

In addition to the 20, three other patients were recruited (2 females, 1 male), but no further news was received about them, and they were therefore excluded.

### 2.3 The Maneuver

The box reports the instructions to the patients for executing the maneuver.

To sum up, the basic elements of the technique are:

1. an active and intense contraction, like the one used when fighting, which corrects incomplete and ineffective contraction (“tension”). It is an isometric contraction, so it involves the simultaneous activation of agonist and antagonist muscles, therefore giving greater generalization.

#### 2.3.1 BOX

##### 2.3.1.1 The Maneuver

The patient was given instructions along the following lines

Phase 1 (Generalized Isometric Sustained Contraction in Apnea, GISCA)

Lying supine (but, especially after sufficient training, also in another position, such as sitting comfortably) inhale deeply until your maximum inspiratory capacity, or close to it; then hold your breath (apnea) and simultaneously carry out a contraction as strong as possible and continuous (a sustained contraction) of as many muscles of the body.

For the initial stages of training, you must contract simultaneously and check in sequence the contraction of abdominal muscles (belly in), buttocks (large glutei), with retroverted pelvis (inclined backwards, pubis forward), pectorals (shoulders lowered; it can help to push the elbows against the support surface), dorsal and neck muscles, limb muscles preferably in extension. This sequence is a suggestion for the initial stages of training; subsequently, you can modify it or, better, be able to activate the simultaneous contraction of all the muscle sectors.

During this contraction (“generalized isometric, sustained”), continuously check the maximum contraction of the various muscle sectors.

Keep counting mentally at the rate of one digit per second.

At the same time, imagine anxiety as something that attacks you, and you have to fight with all your strength; if it is easier for you, imagine or relive the scene of a successful effort to overcome an obstacle or a confrontation in opposition to violence. Focus your attention on this.

Remain in apnea; if you feel the need to breathe, that is to interrupt the apnea, you can take one or more further short inspirations to allow you to hold on a little longer; then, if you can, reinforce the widespread contraction for a moment longer.

Continue therefore for as long as possible: the first few times you will succeed for 10-15 seconds; with practice, you will increase up to 30 seconds and more.

When you really can't resist anymore, catch your breath with a deep breath and relax completely.

Phase 2 (relaxation). Relax at maximum all the muscles that were contracted and continue with deep and slow breaths: 4-7 seconds of inhalation and the same of exhalation, with a moment's pause both at maximum inspiration before moving on to exhalation, and vice versa.

Focus your attention on your breathing, tracking the air moving in and out of your lungs through your airways, and on generalized relaxation. After the initial training, try to choose an image or scene of a pleasant and relaxing experience to focus your attention on while continuing to breathe deeply.

Make the complete relaxation last even for minutes.

Then repeat the whole maneuver from the beginning.

Practice this maneuver regularly, possibly several times a day, until you learn how to perform it without difficulty. Then, during an anxiety attack or a situation in which the anxiety level rises or is expected to rise, the maneuver should be started promptly and repeated until the attack is overcome.

Note that once you've mastered the generalized contraction while lying supine, in bed, or in a comfortable chair, you'll easily be able to perform it while sitting or standing. This will allow you to perform it in a wide variety of situations where you'll have to deal with anxiety.

2. A breath-hold (apnea) on inspiration, which also operates against the dyspnea-hyperpnea typical of anxiety.

A relaxation phase follows with slow, deep breaths. Concentration of attention on the breath, helping to divert attention from anxiety.

Physical effort replaces tension. Effort determines fatigue, which causes the need for refreshment and therefore relaxation as opposed to the state of tension caused by anxiety. The apnea promotes subsequent deep breathing, which also has a relaxing effect.

### 2.4 Procedures

In the practical procedure, the patient's education began with the 2nd phase, that of relaxation, first teaching slow and deep breathing, and then the relaxation of all the muscles. This was done to address first the difficulty that subjects with anxiety often have in regulating their breathing. Once satisfactory execution of this phase had been verified, the patient was instructed on the 1st phase, that of generalized isometric sustained contraction in apnea (GISCA). Satisfactory performance was checked in repeated tests within the same session.

As a home program, the patient was recommended to

(1) Carry out several days of familiarization with the maneuver, performing it even outside of moments of anxiety, until you feel the possibility of carrying it out easily on any occasion.

(2) Use the maneuver in case of an anxiety exacerbation-attack; carry it out repeatedly until, when possible, a satisfactory alleviation of anxiety is obtained. Do it particularly in preparation for exposure to anxiety-provoking situations, such as in cases of social anxiety, particularly performance anxiety.

To evaluate the effect of the maneuver, patients were asked to register the result for each use during anxiety according to 3 criteria.

First of all, a 4-point scale: a score from 0 to 3, with 0 no effect on anxiety, 1 a slight effect, 2 a mild effect, and 3 the maximum effect.

Indeed, scales with many steps (*e.g.*, 0-9) may detect small differences (*e.g.*, only 1 or 2 points) that are practically insignificant, as the objective is to highlight differences that are as certain as possible; small differences do not guarantee a real change. The wording provided is sufficiently clear to avoid the use of images (lines and emoticons) as in classical visual analogue scales.

The task was to record each trial and provide the final evaluation with the average value calculated on at least 3 trials of GISCA and 3 simple relaxation.

This was the first objective to evaluate.

A further objective was to verify whether the use of the complete maneuver or only of the second part, *i.e.*, relaxation, was equally or more effective in reducing anxiety. For this reason, the patient was asked to carry out, on other occasions, only generalized relaxation and to evaluate the comparative effect of the two interventions.

Finally, patients were asked to register if the GISCA phase seemed to facilitate the acquisition of subsequent relaxation; thus, patients had to compare the speed of acquisition of relaxation without and with previous GISCA.

Since the GISCA maneuver is automatically followed by relaxation, the effect of the GISCA maneuver alone cannot be independently assessed.

## 3 RESULTS

Table **1** reports the data of the 20 patients who tried the maneuvers, listed in order of gender (12 females, 8 males) and of recruitment. In addition to the 20, five other patients were recruited (2 females, 3 males), but no further news was received about them, and they were therefore excluded. To summarize: 25 were enrolled for the study, 5 were lost at follow-up, and 20 gave the results shown in detail in Table **1**.

In addition to gender, age, diagnosis, and drug treatment, the table shows the evaluation that patients gave on the degree of improvement in anxiety with the complete maneuver (GISCA+relax, column 4), their comparison between the complete maneuver (GISCA+relax) and relaxation alone (column 5), and finally, whether the GISCA phase appeared to facilitate the subsequent relaxation (column 6).

As stated before, for the first evaluation, a score from 0 to 3 was given, with 0 indicating no effect on anxiety, 1 a slight effect, 2 a mild effect, and 3 a maximum effect.

In one case (#9), a worsening was reported during the same training phase, with abandonment of the test. Two patients (females, #7 and #10) appeared to be stressed by the maneuver and were unable to perform it correctly; they were included as negative results.

As can be seen from the table, the majority of subjects (15 out of 20, that is, 75%) indicated 1 point (7/20, 35%) or 2 points (8/20, 40%) improvement in anxiety with the application of the maneuver (overall average score: 1.15). In 20 cases, the maneuver was effective in 15 cases (75%) and ineffective in 5 cases (25%). Binomial testing demonstrated statistically significant effectiveness (*p* ≈ 0.02; 95% CI: 51–91%). However, if we count as non-responders the 5 cases lost at follow-up (making 10 non-responders), the binomial test indicates a lack of statistically significant effect; therefore, the maneuver results in ineffective treatment in a minority of cases (95% CI: 39–79%).

On occasion of mild attacks, some patients reported total control of anxiety, which would have resulted in a score of 3, but this was not recorded, being occasional and limited to moments of mild anxiety.

The complete maneuver, *i.e.*, the GISCA phase followed by relaxation (GISCA+relax), was considered more effective than generalized relaxation alone in 14 out of 20 patients (70%); the same patients evaluated GISCA as facilitating the subsequent relaxation. Seventeen cases were analyzed to compare the two maneuvers. Maneuver GISCA prevailed in 14 cases (82%) and maneuver relaxation in 3 cases (18%). A binomial test showed that maneuver GISCA prevailed significantly more often than expected by chance (*p* ≈ 0.003; 95% CI: 56–96%).

Three patients (#2, 6, 14) were unable to evaluate or did not provide information on this comparison, while the other three (#7, 9, 10) were those who did not enter the trial due to intolerance of the maneuver.

Table **1** reports the data of the 20 patients who tried the maneuvers, listed in order of gender and of recruitment. The table shows the evaluation that patients gave on the degree of improvement in anxiety with the complete maneuver (GISCA+relax, column 4), then their comparison between the complete maneuver (GISCA+relax) and the relaxation alone (column 5), and finally the facilitation or not that the GISCA phase appeared to have on the subsequent relaxation (column 6).

The complete maneuver provided benefits when faced with various types of anxiety, particularly in the 4 cases of social anxiety, in which subjects were recommended to perform the maneuver before exposure to the anxiety-causing situation.

No one complained of muscular difficulties, pain or cramps, nor any other type of adverse effect.

## 4 DISCUSSION

In the majority of patients, the GISCA maneuver, followed by relaxation, achieved a reduction in anxiety. Again, most patients reported that the GISCA+relax procedure was more effective than generalized relaxation alone and appeared to favor relaxation by speeding up its onset.

Compared to relaxation techniques, GISCA+relax offers the advantage of easier application in acute situations, such as during an anxiety attack or in anticipation of anxiety-provoking events (*e.g.*, performance anxiety).

One might speculate that repeated and prolonged use of generalized isometric muscle contractions presents risks for some people, such as increased blood pressure or additional stress on joints and tendons. However, several studies on “isometric resistance training”, although localized to some muscle groups, have been shown to have an anti-hypertensive effect, due to the vasodilation that follows muscle contraction [25].

The maneuver is essentially a physical exercise. It is not performed against resistance that forces the subject to go beyond their level of adaptation to physical exertion. The subjects perform a generalized contraction and a period of apnea commensurate with their basic capabilities. Indeed, repeating the maneuver simply adds physical exercise to their routine. Beyond the temporary effect, a beneficial action of physical activity on anxiety management is well-demonstrated by many studies [ 26-32]. Isometric contractions seem to have some advantages over isotonic ones in certain conditions from the point of view of athletic performance and are included in some athletic training programs [33-35].

The study of the mechanisms through which physical exercise reduces anxiety suggests several possibilities: apart from molecular mechanisms such as those on BDNF [36, 37] and IGF-1 [38] and others [39], physical effort seems to have a role in inducing tolerance and opposition to anxiety-induced avoidance behavior [39, 40-42], in a way similar to the oppositional physical effort of GISCA.

Breathing regulation is important in controlling anxiety [43-45]. However, some hypothesize that “recurring episodes of apnea are being unconsciously elicited by amygdala activation, resulting in transient spikes in CO2 that provoke fear and anxiety” [46]. In patients in this study, inspiratory apnea does not appear to have had a negative effect on anxiety, probably because the implementation of one or more additional inspirations is allowed, thereby increasing O2 reserve. However, if the hypothesis were valid, it should be kept in mind that the condition to which the hypothesis refers appears to be that of a prolonged arrest or reduction of breathing typical of the immobility corresponding to the state of alert (freezing), which is what GISCA intends to counteract. Furthermore, with GISCA, an inspiratory apnea is implemented, therefore with O2 reserve and with minimal variations in O2 oximetry (as verified several times during the training phase); then, indirectly, minimal CO2 variations. Finally, it should be remembered that apnea causes cerebral vasodilation [47, 48].

The “cognitive” aspect, *i.e.*, the concentration of attention-one more element that could be useful-was not evaluated in these patients because, being outpatients, the training was limited to the most relevant elements of the maneuver. Likely, the inclusion of a strong concentration of attention and guided imagery [49, 50] could lead to further improvement in the effectiveness of the maneuver.

The open methodology used had the advantage of evaluating feasibility and verifying the effectiveness of the simple procedure, which can be implemented in a normal outpatient clinical practice for patients with anxiety disorders or, in any case, during moments of anxiety.

The data obtained appear to indicate that the maneuver can be an aid in controlling anxiety. For this reason, we suggest proposing it.

## 5 LIMITATIONS

This study represents a practical open evaluation, where each patient served as her/his own control. This aimed to provide an initial insight into the maneuver’s clinical feasibility and effectiveness when applied by outpatients in common clinical practice. More rigorous evaluation, with extended training, will be necessary to confirm the maneuver’s true efficacy and to refine technical variations if needed. Patient-reported data were not systematically documented *via* written reports or objective instruments; therefore, accuracy may vary. Nonetheless, patients who reported improvement continued performing the maneuver, indicating a perceived benefit. This is indicative against a placebo effect: since the maneuver requires a certain effort and commitment, it would not be chosen by the patient if perceived as ineffective.

In the future, electrophysiological recording of various parameters could be performed for a better understanding of the effects. Moreover, the addition of “cognitive”/“guided imagery” aspects deserves further evaluation. An evaluation of the response in cases of anxiety within specific pathology groups may be useful.

Finally, the awareness of being able to address anxiety with non-pharmacological interventions (*e.g.*, as-needed use of benzodiazepines) might have positively influenced subjective evaluation. However, this might be considered a positive factor in the practice of treating patients, given the simplicity of use as well as the possibility of a beneficial physical effect.

## CONCLUSION

This new somatic behavioral approach opposes anxiety, possibly by activating a primordial defense mechanism, as hypothesized when it was conceived. Our first results indicate that the maneuver can help in controlling anxiety. Since it can be acquired by patients with sufficient simplicity, it is suggested that it be considered as an alternative to other somatic techniques or to the as-needed use of drugs.

## Figures and Tables

**Table 1:** Effects of the maneuver as reported by patients.

Gender (F, M) Age (Years)	Diagnosis	Ongoing Medications	GISCA+relax. Effectiveness (0-3 points)	GISCA+relax. *vs* Relaxation only	GISCA Facilitating Relaxation
1.F, 63	Multiple phobias	Escitalopram	2	Better	Yes
2. F, 40	Panic disorder	Escitalopram	1	Not evaluated	Not evaluated
3. F, 60	Obsessive-compulsive dis., anxiety	Clomipramine	1	Better	Yes
4. F, 23	Multiple phobias	Sertraline	1	Better	Yes
5. F, 21	Generalized anxiety dis., phobias	Venlafaxine	2	Better	Yes
6. F, 58	Anxiety, panic dis.	Fluoxetine	0	Not evaluated	Not evaluated
7. F, 56	Anxiety, depression	Escitalopram	0	Worse	No
8. F, 28	Generalized anxiety dis.	Fluoxetine	2	Better	Yes
9. F, 40	Panic dis.	Venlafaxine	0	Worse	No
10. F, 64	Anxiety, depression	Sertraline	0	Worse	No
11. F, 17	Social anxiety	Escitalopram	2	Better	Yes
12. F, 44	Social anxiety	Escitalopram	2	Better	Yes
13. M, 60	Separation anxiety	Escitalopram	2	Better	Yes
14. M, 29	Bipolar	Lithium, valproate	0	Not evaluated	No
15. M, 56	Anxiety, depression	Escitalopram	1	Better	Yes
16. M, 18	Social anxiety	Escitalopram	2	Better	Yes
17. M, 54	Bipolar dis, anxiety	Mirtazapine, lamotrigine	1	Better	Yes
18. M, 30	Social anxiety	Clomipramine	2	Better	Yes
19. M, 40	Bipolar dis, anxiety	Escitalopram, amisulpride	1	Better	Yes
20. M, 28	Anxiety, depression	Escitalopram	1	Better	Yes

**Abbreviation:** GISCA= generalized isometric sustained contraction in apnea. Relax= relaxation.

## Data Availability

The data and supportive information is available within the article.

## LIST OF ABBREVIATIONS

GISCA: Generalized Isometric Sustained Contraction in Apnea
PMR: Progressive Muscle Relaxation
BDNF: Brain-Derived Neurotrophic Factor
